# Supplementary material for: Splice-Junction-Based Mapping of Alternative Isoforms in the Human Proteome
Source: Cell Rep. Author manuscript; Available in PMC 2020 Jan 15. (PMC6961840; doi:10.1016/j.celrep.2019.11.026)

A

sp|O14974|MYPT1_HUMAN|ENSG00000058272|A3SS2|1903|chr12|79781862|79779351|−2|r49|T4
ISEMEEELKR q value: 4.6505e−05 Tr_novel:TRUE RefSeq_Novel:TRUE
Search result spec prec mz: 421.8809 Actual spec prec mz: 421.88094
Fragments matched per AA: 2.4 Proportion of top 20 peaks matched: 0.4

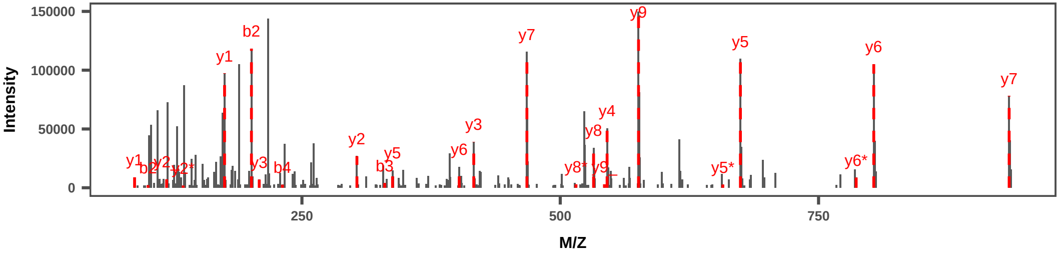

B

Scatterplot of predicted elution time
Fitting R2: 0.869
Novel peptide residual Z score: −0.888
Number of peptides: 2810

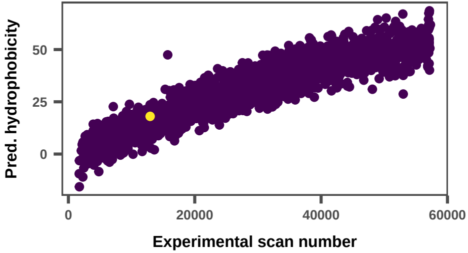

C

Distributions of residuals from best−fit line
of predicted RT vs Expt. scan number
Line: Z score of novel peptide
Z: −0.888

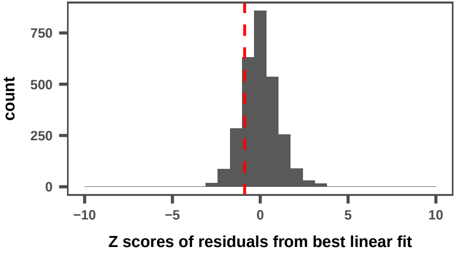

Supplement: 2 [file NIHMS1546469-supplement-2.zip › DF1/PXD010154/Lung/Lung_9_PPP1R12A_ISEMEEELKR.pdf]
